# The Neuroinflammatory Response in ALS: The Roles of Microglia and T Cells

**DOI:** 10.1155/2012/803701

**Published:** 2012-05-15

**Authors:** Coral-Ann Lewis, John Manning, Fabio Rossi, Charles Krieger

**Affiliations:** ^1^Department of Biomedical Physiology and Kinesiology, Simon Fraser University, Burnaby, BC, Canada V5A 1S6; ^2^The Biomedical Research Centre, University of British Columbia, Vancouver, BC, Canada V6T 1Z3; ^3^Division of Neurology, Department of Medicine, Neuromuscular Disease Unit, VHHSC, Vancouver, BC, Canada V5Z 1M9

## Abstract

Amyotrophic lateral sclerosis (ALS) is a fatal neurodegenerative disease characterized by upper and lower motoneuron death. Mutations in the gene for superoxide dismutase 1 (SOD1) cause a familial form of ALS and have been used to develop transgenic mice which overexpress human mutant SOD1 (mSOD) and these mice exhibit a motoneuron disease which is pathologically and phenotypically similar to ALS. Neuroinflammation is a pathological hallmark of many neurodegenerative diseases including ALS and is typified by the activation and proliferation of microglia and the infiltration of T cells into the brain and spinal cord. Although the neuroinflammatory response has been considered a consequence of neuronal dysfunction and death, evidence indicates that manipulation of this response can alter disease progression. Previously viewed as deleterious to neuronal survival, recent reports suggest a trophic role for activated microglia in the mSOD mouse during the early stages of disease that is dependent on instructive signals from infiltrating T cells. However, at advanced stages of disease, activated microglia acquire increased neurotoxic potential, warranting further investigation into factors capable of skewing microglial activation towards a neurotrophic phenotype as a means of therapeutic intervention in ALS.

## 1. Introduction

Neuroinflammation is a pathological hallmark of many neurodegenerative diseases including Alzheimer's disease (AD), Parkinson's disease (PD), and amyotrophic lateral sclerosis (ALS). It is characterized by the activation and proliferation of microglia (microgliosis) and the accumulation of infiltrating T lymphocytes at sites of neurodegeneration. Although often considered a consequence to neuronal injury and degeneration, the neuroinflammatory response can have protective or deleterious effects on neuronal survival. These disparate effects are elicited by the heterogeneous activation programs of microglia, which in turn are dictated by their surrounding microenvironment and by infiltrating T cells.

## 2. Amyotrophic Lateral Sclerosis and the mSOD Mouse Model

Typically diagnosed during the fifth decade of life, amyotrophic lateral sclerosis (ALS) is a fatal neurodegenerative disease characterized by the degeneration of motoneurons in the brainstem and spinal cord and loss of descending motor tracts. Clinical manifestations of ALS include muscle weakness, spasticity, muscle atrophy, and advancing paralysis that culminates in respiratory failure, the usual cause of death in affected patients. ALS is a disease primarily of sporadic etiology with a plethora of aberrant physiological processes implicated in its pathogenesis including excitotoxicity, oxidative damage, the formation protein aggregates, and mitochondrial dysfunction [1]. A pathological hallmark of sporadic ALS is the presence of cytoplasmic ubiquitinated protein inclusions in affected areas of the brain and spinal cord that are predominantly composed of the TDP-43 (transactive response DNA-binding protein 43), an RNA/DNA-binding protein normally found in the nucleus [2].

A small fraction of cases (~10%) termed familial ALS (fALS) are due to a variety of genetic mutations, with 20% of fALS cases due to dominantly inherited mutations in superoxide dismutase 1 (SOD1). SOD1 is a ubiquitously expressed, 32 kDa homodimeric cytosolic protein that catalyzes the dismutation of superoxide, a by-product of cellular respiration, to hydrogen peroxide. To date, over 125 different mutations that span the entire genomic sequence and protein structure of SOD1 have been identified as causing ALS [3]. In 1994, Gurney et al. [4] developed transgenic mice that overexpress mutant SOD1 (mSOD) and develop a progressive motoneuron degeneration resembling ALS, including cytoplasmic mislocalization of TDP-43 at end-stage of disease [5]. However, after years of investigation, the pathogenic basis of mSOD remains elusive. The majority of SOD1 mutants retain at least partially normal enzyme activity and ablation of the murine SOD1 gene does not culminate in motoneuron pathology [6], indicating that the pathogenic nature of mSOD is through a toxic gain of function rather than a loss of function. Several pathogenic mechanisms of mSOD have been suggested including an increased propensity to form intracellular aggregates, aberrant enzyme activity, ER stress, mitochondrial dysfunction, and glial dysfunction contributing to motoneuron death [7].

An added complexity to mSOD pathogenicity is experimental evidence indicating that motoneuron death in the mSOD model is a noncell autonomous event. Although mSOD expression restricted to neurons is sufficient to cause motoneuron death if expressed at adequate levels [8], mSOD expression in surrounding astrocytes and microglia influences the rate of progression of neurodegeneration. Experiments in which mSOD expression in microglia was reduced [9] or ablated [10] prolonged disease duration and extended survival in mSOD mice but did not affect the time of disease onset. Similarly, the establishment of wild-type astroglial pools via the transplantation of astroglial precursors into the mSOD spinal cord resulted in prolonged survival in mSOD mice [11]. Notably, restricted mSOD expression in astrocytes or microglia is not sufficient to cause dysfunction in wild-type neurons [12]. Together these results suggest that the onset of neurodegeneration in the mSOD mouse is due to mSOD expression in motoneurons but that the rate of disease progression is influenced by mSOD expression in surrounding microglia and astrocytes.

## 3. Microglia: CNS Resident Macrophages

Within the CNS, populations of macrophages can be distinguished based on their anatomical location. Perivascular macrophages lie between the basal lamina of blood vessels and the glia limitans while meningeal macrophages lie within the leptomeninges that surround the CNS. Microglia are considered the CNS tissue-resident macrophage population and are found within the parenchyma of the CNS. These cells possess a characteristic stellate morphology, with long sinuous processes extending from a round cell body. In their quiescent state, microglia are highly dynamic cells, surveying their surrounding microenvironment through the constant extension and retraction of their processes; it is estimated that the entire extracellular space of the CNS is surveyed every few hours [13].

The phenotype of resting microglia differs from that of other populations of tissue macrophages, being more similar to that of immature myeloid cells; microglia express only low levels of CD45, major histocompatibility complexes (MHCs), and are poor antigen presenting cells (APCs; [14]). The downregulated phenotype of microglia, along with a lack of a conventional lymphatic system and the segregation of the brain parenchyma from peripheral blood by the blood-brain barrier, provides the CNS with a status of immune privilege. This immune specialization enables the suppression and strict regulation of immune responses that could damage surrounding neurons that have only limited regenerative potential. This should not suggest that the CNS is not immune competent, as foreign pathogens, proinflammatory cytokines, or neuronal injury induces microglial activation characterized by morphological alterations including the retraction and thickening of processes and hypertrophy of the cell body [15]. Although these morphological changes are stereotypical with regards to microglial activation, as with other macrophage populations, the phenotype of activated microglia can be highly variable.

Often likened to a double-edged sword in the literature, activated microglia can produce substances that are either beneficial or toxic to surrounding neurons. M1- (classically) activated microglia exhibit a proinflammatory phenotype characterized by the production of interleukin- (IL-) 1*β* and tumor necrosis factor *α* (TNF-*α*) and increased release of reactive oxygen species and nitric oxide through upregulated expression of NADPH oxidase and inducible nitric oxide synthase (iNOS), respectively (Table 1). *In vitro*, cocultured microglia and neurons treated with lipopolysaccharide (LPS), a potent stimulus for M1 activation of macrophages, result in increased microglial production of nitric oxide (NO) and reactive oxygen species (ROS), as well as increased levels of extracellular glutamate which culminates in the excitotoxic death of neurons [16]. Treatment of cultured microglia using IL-4 results in an M2- (alternatively) activated phenotype typified by the enhanced expression of anti-inflammatory cytokines (e.g. IL-10) that dampen inflammation and lead to the release of neurotrophic factors (e.g., IGF-1, GDNF) that support neuronal survival (Table 1). Another feature distinguishing M1 and M2 activation programs is the metabolism of L-arginine; in M1-activated macrophages and microglia, upregulation of iNOS converts L-arginine to NO, while in M2-activated macrophages it converts L-arginine to L-ornithine (Table 1, [17]).

Neurons play an integral role in regulating microglial activation by expressing membrane bound and soluble mediators that enhance microglial production of anti-inflammatory cytokines and neurotrophins. For example, CD200 is a glycoprotein expressed by neurons and its cognate receptor (CD200R) is expressed by all myeloid cells including microglia. In the CNS of mice deficient for CD200R, microglia were observed to possess activated morphologies under steady-state conditions and exhibited an enhanced response following facial nerve axotomy compared to similarly treated wild-type mice [18] suggesting that neuronal expression of CD200 regulates microglial activation. A second example of how neurons regulate microglial function is through the chemokine fractalkine (CX3CL1) which is expressed on neuronal cell membranes. Following proteolytic cleavage, CX3CL1 is released into the extracellular milieu and affects microglia exclusively as microglia are the only cells within the CNS that express the fractalkine receptor (CX3CR1). CX3CR1^−/−^ mice exhibit dysregulated microglial responses following peripheral injection of LPS, while CX3CR1 ablation in mSOD mice results in increased levels of neuronal loss [19]. Neuronal communication with microglia keeps inflammatory responses in check, preventing neuronal damage by aberrantly activated microglia.

## 4. T Cells

T cells are the central players in adaptive immunity and can be divided into different subsets based on the expression of cell surface molecules and function (Table 2). Cytotoxic T cells (CTLs) express CD8 and are capable of inducing apoptosis in cells through the expression of Fas ligand and through the exocytosis of perforin and granzymes [20]. The Fas ligand (CD95L) expressed on CD8^+^ T cells interacts with Fas (CD95) expressed on host cells to induce the downstream activation of caspases, culminating in the apoptosis of host cells. Perforin induces the formation of pores on the target cell membrane, which can result in osmotic cell lysis and provides a means of entry for secreted granzymes [21].

T lymphocytes expressing CD4 include helper T cells (Th) that are further classified according to cytokine production profiles and effector functions and T-regulatory cells (Tregs; Table 2). Compared to CTLs, CD4^+^ T cells have only limited ability to directly kill cells; they do not express Fas ligand or secrete granulysin and function mainly to activate and regulate the activity of other cells involved in the immune response, including macrophages and microglia [22]. For example, Th1 and Th17 cells can promote M1 macrophage activation through the secretion of the proinflammatory cytokines IL-1 and IL-17, respectively, while Th2 cells secrete cytokines that antagonize proinflammatory mediators and are capable of skewing macrophage activation towards an M2 phenotype through the secretion of IL-4 [23]. Regulatory T cells (Tregs) are characterized by the expression of CD4, CD25, CD62L, CD103, CD152, and the FoxP3 transcription factor which is essential for obtaining the Treg phenotype [21]. For each adaptive immune response launched, a corresponding regulatory response is elicited and mediated by Treg cells that function to regulate the type and level of immune activation [23].

Naive T cells are activated upon recognition and binding of antigen specific to their expressed T-cell receptor; differentiation to a specific effector subtype is determined by the local microenvironment. For CD4^+^ T cells, antigen is presented on MHC class II molecules on the membranes of APCs, typically dendritic cells, and activated macrophages. CD8^+^ T cells recognize antigen presented on MHC class I molecules which are expressed on the membranes of all nucleated cells with the exception of neurons and other cell populations within the CNS; however, under neuroinflammatory conditions, neurons upregulate their MHC class I expression, making them potential targets for CTLs [24]. Notably, after the phagocytosis of foreign pathogens or neuronal debris following injury or degeneration, macrophages can cross-present antigens on MHC class I molecules to CD8^+^ T cells, resulting in their activation and potential reactivity to neuronal cells [20]. Neuronal antigen-specific CD8^+^ T cells must first be activated within secondary lymphoid organs before migration and extravasation into the CNS. This may be accomplished through antigen drainage of cerebrospinal fluid into the cervical lymphatics or through the migration of APCs residing in the perivascular space to lymph nodes [20]. For both CD4^+^ and CD8^+^ T cells, a secondary independent signal elicited through the binding of molecules present on the membranes of host cells is essential for activation and clonal expansion; this secondary signal from APCs may be stimulatory or inhibitory. The types of costimulatory or coinhibitory molecules expressed by APCs confer the nature of their functional activation states, while the density of costimulatory and coinhibitory molecules on T-cell membranes dictates the functional outcome of T-cell activation [25]. In the absence of costimulation, T cells enter a state of anergy and are incapable of activation upon subsequent antigen recognition by their T-cell receptor [21].

Activated T cells are capable of extravasating into the CNS where they perform immune surveillance, and in the steady state, variable numbers of T cells are present within the parenchyma of the CNS [26]; however, very few if any CTLs are observed in the healthy CNS [27]. The healthy CNS parenchyma lacks resident dendritic cell populations but these cells are present within the meninges and perivascular spaces [28], and upon activation, microglia increase their expression of MHC class II molecules, becoming proficient APCs [29].

Once T cells are present within the extracellular space of the CNS, resident parenchymal cells including microglia and neurons are capable of mediating T-cell responses through cell-cell contact, representing a further source of protection from rogue immunological responses. All cells within the parenchyma of the CNS constitutively express Fas-ligand, which upon contact with activated Fas-expressing CD8^+^ T cells surveying the CNS for their cognate antigen, induces the CD8^+^ T-cell apoptosis [30]. Microglia are also capable of modulating T-cell responses as they constitutively express B7 homolog 1 (B7-H1) and increase their expression in the presence of proinflammatory cytokines IL-1 and interferon-*γ* (IFN-*γ*; [31]). B7-H1 interacts with the programmed-death receptor 1 (PD-1) expressed on T cells, inhibiting T-cell activation and cytokine secretion [20]. These factors contribute to CNS-associated immune privilege by preventing aberrant inflammatory reactions and the consequent neuronal injury they could impart.

## 5. Neuroinflammatory Response in the mSOD Mouse Model of ALS

Neurodegenerative diseases including Parkinson's disease, AD, and ALS are characterized by the death of specific populations of neurons accompanied by a neuroinflammatory response that is characterized by microglial activation and T-cell infiltrates being at affected regions. Significant levels of microgliosis have been observed in the spinal cord of ALS patients at autopsy, with T-cell infiltrates found in close proximity to the corticospinal tract [32, 33] as well as in other affected brain regions [34]. Histological examination of CNS tissue from ALS patients is typically limited to advanced stages of disease. However, studies using PET scanning and other imaging techniques permit evaluation of ALS patients at various stages of their disease. Turner et al. [35] administered the radioligand [11C]-(R)-PK11195 to ALS patients, which binds the translocator protein (formerly known as the peripheral benzodiazepine receptor) that is highly expressed on mitochondria of activated, but not resting microglia. This enabled PET detection of cerebral microglial activation *in vivo* over the disease course. Widespread microglial activation was observed in the motor cortex, pons, dorsolateral prefrontal cortex, and thalamus where the extent of microgliosis was positively correlated with the severity of ALS [35].

Notably, patients suffering from sporadic ALS have been reported to have increased levels of circulating inflammatory (CD16^+^) monocytes in peripheral blood [36], which correlated well with increased levels of plasma LPS [37], a potent inducer of M1 activation in macrophages. These results indicate that the inflammatory response associated with ALS is not limited to the CNS, with systemic immune activation also being observed and potentially influencing disease progression. Furthermore, recent reports by Swarup et al. demonstrated that mRNA levels of TDP-43 and the p65 subunit of nuclear factor *κ*B (NF-*κ*B), a transcription factor involved in the expression of proinflammatory mediators, are upregulated in the spinal cords of ALS patients [38]. When cultured microglia engineered to overexpress TDP-43 were treated with LPS, increased levels of proinflammatory cytokines and neurotoxic factors were produced compared to wild-type microglia [38]. Together, the increased levels of plasma LPS and TDP-43 observed in ALS patients indicate widespread inflammation and suggest that modulation of the inflammatory response may represent an avenue of therapeutic intervention.

As the mSOD mouse model recapitulates many aspects of the neuroinflammatory response observed in ALS patients, this model enables an in-depth analyses of neuroinflammation at different stages of disease. In the mSOD mouse, increased numbers of activated microglia are observed at early presymptomatic stages of disease, and with disease progression to end-stage, microglial numbers in the lumbar spinal cord increase further by nearly 2-fold [39, 40]. Increased numbers of T cells are found in the lumbar spinal cord of mSOD mice beginning at presymptomatic stages and increasing with disease progression to symptomatic and disease end-stage, where T-cell numbers are 10-fold higher than of controls (Lewis unpublished data; [40, 41]). Phenotypical analysis indicated that T cells populating the mSOD spinal cord were limited to the CD4^+^ subsets until disease end-stage at which point 40% of T cells were CD8^+^ CTLs ([41]; Lewis unpublished data). 

Although neuroinflammation is often considered a consequence rather than a cause of neurodegeneration in ALS patients and in the mSOD mouse model, several studies have demonstrated that modulation of the inflammatory response in mSOD mice alters disease progression [40–44]. Given that reports that anti-inflammatory drugs including minocycline slowed the rate of disease progression and extended survival times in mSOD mice [42–44] and because mSOD-expressing microglia exhibit enhanced neurotoxicity when treated with LPS [45], it was postulated that microgliosis in the mSOD mouse contributed to motoneuron degeneration. However, experiments in which the proinflammatory cytokine TNF-*α* was ablated in mSOD mice [46] or where the proliferation of microglia was blocked [47] had no effect on the rate of disease progression, suggesting that microgliosis does not exacerbate neurodegeneration in the mSOD mouse mode.

While previous research has focused on the potential neurotoxicity of activated microglia in the mSOD mouse model, recent work has raised the hypothesis that activated microglia might confer neuroprotection. Phenotypical analysis of microglia in mSOD mice using RT-PCR demonstrated that the expression of the neurotrophic factor IGF-1 by microglia increased with disease progression, as did the expression of the anti-inflammatory IL-1R antagonist which binds to the IL-1 receptor, blocking IL-1 binding and downstream proinflammatory signalling; levels of the proinflammatory cytokine TNF-*α* did not change with disease progression [40]. Recent work by Beers et al. [48] supports a neuroprotective role for microglia until the end-stage of disease, at which point levels of proinflammatory cytokine IL-1*β* and TNF-*α* increase, as do levels of NADPH oxidase [48]. These observations suggest that during initial stages of disease in mSOD mice, microglia exhibit an M2 phenotype supporting neuronal survival. However, as the disease advances, microglial activation becomes skewed towards an M1 phenotype, although the physiological mechanisms eliciting this switch in activation have not been elucidated.

Investigations into the role of T cells in neuroinflammation in the mSOD mouse suggest that these cells influence the phenotypic profile of activated microglia. In two independent studies, ablation of T cells in mSOD mice was achieved by crossing these mice with a TCR^−/−^ strain [40] or with an RAG2^−/−^ strain [41], and disease progression was accelerated in the mSOD mice [49]. In both studies, microglial morphological activation in mSOD mice lacking functional T cells was reduced; however, levels of M1 functional markers such as TNF*α* and iNOS were increased while markers of alternative activation such as IGF-1, GDNF-1, TGF-B, and IL-4 were reduced [41]. To further identify which T-cell subsets were capable of affecting disease course, the mSOD mice were crossed onto a strain lacking only functional CD4^+^ T cells [41]. The observed result was similar to that demonstrated by the studies in which all T cells were ablated, indicating that CD4^+^ T cells in the mSOD spinal cord function to modulate microglial activation and skew it towards an M2 neuroprotective phenotype [41]. Banerjee et al. [50] further refined these observations by comparing the effects of adoptive transfer of activated CD4^+^CD25^+^ Treg cells and CD4^+^CD25^−^ Teff cells harvested from wild-type mice on disease progression in mSOD mice. The transfer of Treg cells delayed disease onset while transfer of Teff cells prolonged disease progression and the duration of survival [50]. Notably, CD8^+^ T cells have not been observed in mSOD spinal cord until disease end-stage (Lewis unpublished data; [40]), a time point that corresponds temporally with reduced numbers of CD4^+^CD25^+^ and CD25^+^ Treg cells in mSOD spinal cord and the skewing of microglial phenotypes towards M1 activation [48].

Findings from these studies suggest that exploiting the neurotrophism of alternatively activated microglia, rather than dampening microglial activation generally, may have some therapeutic benefit in ALS; however, molecular targets enabling this manipulation remain elusive. Recently Beers et al. [51] demonstrated that the passive transfer of CD4^+^ Tregs into mSOD mice extended the stable phase of disease progression and survival times, suggesting that manipulation of the microglial response through the adoptive transfer of Treg cells or pharmaceutical agents that potentiate M2 activation in microglia may have therapeutic value. In fact, Neuraltus Pharmaceuticals (Palo Alto, CA) is currently conducting phase II clinical trials in patients suffering from ALS, PD, and AD using NP100, a pharmaceutical drug designed to skew macrophage activation towards an M2 phenotype to determine its efficacy in prolonging disease duration.

## 6. A Role for Bone-Marrow-Derived Microglia

Pharmacological treatments for ALS have largely been ineffective at slowing the disease process, in part because the blood-brain barrier prevents the transmission of the majority of drugs from the blood into the CNS. This has spurred investigations into alternative therapeutic modalities for the treatment of ALS and other neurodegenerative diseases. Microglia are members of the mononuclear phagocyte system which also includes hematopoietic progenitors, blood monocytes, dendritic cells, and other populations of tissue macrophages [52]. Under inflammatory conditions and to a lesser extent during the steady state, circulating monocytes are recruited to tissue compartments where they extravasate and differentiate into macrophages. Although it has been well established that macrophage populations in nonneuronal tissues are maintained to a variable degree through the recruitment of monocytes [53], evidence indicates that only under certain conditions myeloid cells contribute to the maintenance of microglial populations. This highlights the potential for these cells to function as vehicles to transport neurosupportive substances into the diseased CNS.

Investigations into the migration of bone-marrow-derived cells (BMDCs) into the CNS often employ bone marrow (BM) chimeric mice, typically created by exposing rodents to myeloablative levels of radiation followed by the adoptive transfer of labelled BM cells. The results of these studies suggest that while BMDCs contribute to the maintenance of meningeal and perivascular macrophage populations within the CNS, BMDCs make only limited contributions to the parenchymal microglial pool [54–57]. However, in BM chimeric models of neurodegenerative disease including PD, AD, and ALS, increased numbers of BMDCs are observed at sites of neurodegeneration, suggesting BMDCs home to and/or expand at affected sites.

A caveat associated with the irradiation-BM reconstitution protocol employed to create BM-chimeric mice is that it introduces two confounding variables. First, irradiation elicits a widespread inflammatory response including increased levels of cytokines and chemokines within the CNS [58] and has been shown to induce apoptosis of endothelial cells in the rat spinal cord-blood barrier [59]. Secondly, the injection of whole BM into the circulation of mice introduces BM progenitor populations into the blood that would under normal physiological circumstances not enter the circulation [60]. Indeed, studies employing chimeric mice created through parabiosis, a surgical technique in which the vascular systems of two genetically distinct mice are joined, demonstrated that in the absence of irradiation and the injection of BM precursor populations into the circulation, BMDCs do not appreciably accumulate within the healthy CNS, or in models of neuronal injury and neurodegenerative disease [58, 60, 61]. Recent work by Ajami et al. [62] suggests that in irradiated BM-chimeric mice, hematopoietic precursors contribute to microglial populations while blood monocytes infiltrating the CNS during experimental autoimmune encephalitis (EAE) in chimeric mice represent a transient population of CNS-associated macrophages that turnover upon disease resolution. Therefore, in order to improve the clinical potential of BMDCs as treatment vehicles in neurodegenerative disease, the cell populations within BM capable of infiltrating the CNS and contributing to microglial pools and factors enabling this migration must be identified.

## 7. Conclusion

Once considered a consequence of neuron death in chronic neurodegenerative disease, neuroinflammation is now recognized to influence disease progression in ALS and the mSOD mouse model. While microglial activation and T-cell infiltration have previously been implicated in exacerbating pathological processes and contributing neuron death in the mSOD mouse, experimental evidence has demonstrated that microglial activation together with the infiltration of instructive T cells has trophic effects on surrounding neurons until late stages of disease. Further investigations into phenomena that induce this phenotypical switch in activated microglia could potentially enable the exploitation of microglial neurotrophism and provide future therapeutic benefits.

## Figures and Tables

**Table 1:** Macrophage activation programs can be distinguished by the associated release of cytokines, arginine metabolism, secreted release of mediators, and antigenicity.

	M1	M2
Cytokines released	TNF-*α*, IL-1*β*, IL-6, IL-12, IL-23	IL-10, IL-4, IL-13, TGF-*β*
Arginine metabolism	iNOS *→* NO	arginase 1*→* L-ornithine
Other secreted mediators	NO, ROS	Neurotrophics (GDNF, IGF-1)
Antigenicity	IL-1R, CCR7	IL-1Ra, CD150, CD14, CD163

**Table 2:** The categorization of T cells into subsets is based on cell antigenicity, cytokine profile, and effector function.

	Antigenicity	Cytokine profile	Effector function
Th1	CD4^+^	IL-2, TNF-*α*, IFN-*γ*	M1 macrophage activation
Th2	CD4^+^	IL-4, IL-10, IL-6, IL-13	Downregulation of M1 macrophage activation
Th17	CD4^+^	IL-17	M1 macrophage activation
Treg	CD4^+^CD25^+^FoxP3^+^	IL-4, IL-10, TGF-*β*	Damping of proinflammatory response
CTL	CD8^+^	TNF-*α*, IFN-*γ*	Elimination of infected cells
